# Regulator of nonsense transcripts 3B is a prognostic biomarker and associated with immune cell infiltration in lung squamous cell and hepatocellular carcinoma

**DOI:** 10.1007/s12672-024-01369-3

**Published:** 2024-09-27

**Authors:** Pengcheng Li, Mi Zhou, Xiaoli Gan, Chaoyi Yuan, Ganxun Li, Guan-nan Jin, Ze-yang Ding

**Affiliations:** 1 Hepatic Surgery Center, Clinical Medicine Research Centre for Hepatic Surgery of Hubei Province, and Hubei Key Laboratory of Hepato-Pancreato-Biliary Diseases, Tongji Hospital, Tongji Medical College, Huazhong University of Science and Technology, Wuhan, Hubei 430030 People’s Republic of China; 2 Department of Internal Medicine, Union Hospital, Tongji Medical College, Huazhong University of Science and Technology, Wuhan, Hubei 430071 People’s Republic of China

**Keywords:** RENT3B, Pan-cancer, Immune infiltration, Prognosis analysis

## Abstract

**Purpose:**

The characteristic of RENT3B in cancer remains ambiguous. We aimed to study the relationship between RENT3B and immune infiltration in liver hepatocellular carcinoma (LIHC) and lung squamous cell carcinoma (LUSC).

**Patients and methods:**

We investigated the expression levels of RENT3B using ONCOMINE and TIMER databases, and assessed the interrelationship between RENT3B expression and survival using PrognoScan, GEPIA, and Kaplan–Meier plotter. Additionally, we examined the association between RENT3B and immune cells in the tumor microenvironment (TME), as well as markers of immune cells, using TIMER. Subsequently, we performed prognostic analysis based on the expression level of RENT3B within specific immune cell subgroups. Furthermore, we evaluated the promoter methylation profile of RENT3B between tumor and normal tissues in LIHC and LUSC using the DNMIVD database.

**Results:**

RENT3B exhibited increased levels in both in LIHC and LUSC. High RENT3B expression was associated with unfavorable prognosis in LIHC, whereas it indicated a beneficial prognosis in LUSC. In LIHC, the expression of RENT3B positively correlated with immune infiltration levels of B cells, CD4 + T cells, CD8 + T cells, neutrophils, macrophages, and dendritic cells. However, in LUSC, the expression of RENT3B showed a negative correlation with immune infiltration levels of B cells, CD8 + T cells, neutrophils, macrophages, and dendritic cells. RENT3B exhibited positive correlations with 42 immune markers in LIHC, while it displayed negative associations with 10 immune markers in LUSC. Despite variations in immune cell enrichment and reduction subgroups, high RENT3B expression consistently indicated poor prognosis in LIHC, whereas it remained favorable in LUSC. Additionally, there were no significant differences observed in RENT3B promoter methylation between tumor and normal tissues in both LIHC and LUSC.

**Conclusion:**

RENT3B can affect the overall tumor prognosis and is associated with immune infiltration, especially in LIHC and LUSC. Consequently, RENT3B can become a prognostic biomarker for LIHC and LUSC.

**Supplementary Information:**

The online version contains supplementary material available at 10.1007/s12672-024-01369-3.

## Introduction

The occurrence of tumors is intricately linked to the physiological state of cells and the stroma in the tumor microenvironment (TME). Moreover, tumor progression is heavily reliant on specific characteristics of the TME, which can significantly influence both prognosis and chemotherapy efficacy [1]. The tumor microenvironment encompasses a diverse array of cells, with infiltrating immune cells comprising a substantial proportion. These include T and B lymphocytes, natural killer cells, and tumor-associated macrophages [2]. While it has been conventionally believed that immune cells have anti-tumor effects, recent research has revealed a contrasting perspective. Immune cells that have infiltrated the tumor microenvironment can paradoxically aid in preventing cancer cell eradication while also contributing to cancer development [2, 3]. For instance, tumor-associated macrophages (TAMs) can assist tumor cells through various mechanisms, including immune evasion, tumor angiogenesis, and metastasis [4–7]. The degree of immune infiltration exerts a crucial impact on tumor progression and patient prognosis. Considering the factors influencing immune infiltration, early identification of these factors may potentially delay tumor progression and enhance patient prognosis.

The RENT3B gene encodes a protein that is a component of a spliced multiprotein complex involved in the nuclear export of mRNA and mRNA monitoring. This protein binds to mRNA and remains bound after nuclear export, acting as a nuclear-cytoplasmic shuttle protein. mRNA monitoring plays a critical role in identifying mRNA with truncated open reading frames and initiating nonsense-mediated mRNA decay (NMD) process [8]. Most research on RENT3B has focused on NMD, with numerous studies exploring its association with diseases such as intellectual disability, autism, and schizophrenia [9–11]. However, there is a scarcity of studies investigating the involvement of RENT3B in tumor, particularly its relationship with immune infiltration. This article aims to provide a brief overview of the connection between RENT3B and tumor.

In the present study, we investigated the expression of RENT3B in various cancers using the ONCOMINE and Tumor Immune Estimation Resource (TIMER) databases. [12, 13]Additionally, we explored the prognostic value of RENT3B expression in pan-cancer by utilizing the PrognoScan, GEPIA, and Kaplan–Meier plotters databases [14–17]. Notably, our analysis revealed distinct prognostic patterns of RENT3B in LIHC and LUSC. Furthermore, we examined the relationship between RENT3B and immune infiltration levels in these two tumor types using the TIMER database. Additionally, we investigated the promoter methylation profile of RENT3B in these cancers by analyzing the DNMIVD database [18]. Our findings suggest that RENT3B may significantly impact the prognosis of cancer patients through its interaction with immune cells within the tumor microenvironment.

## Results

### The mRNA expression level of RENT3B in pan-cancer

**Fig. 1:**
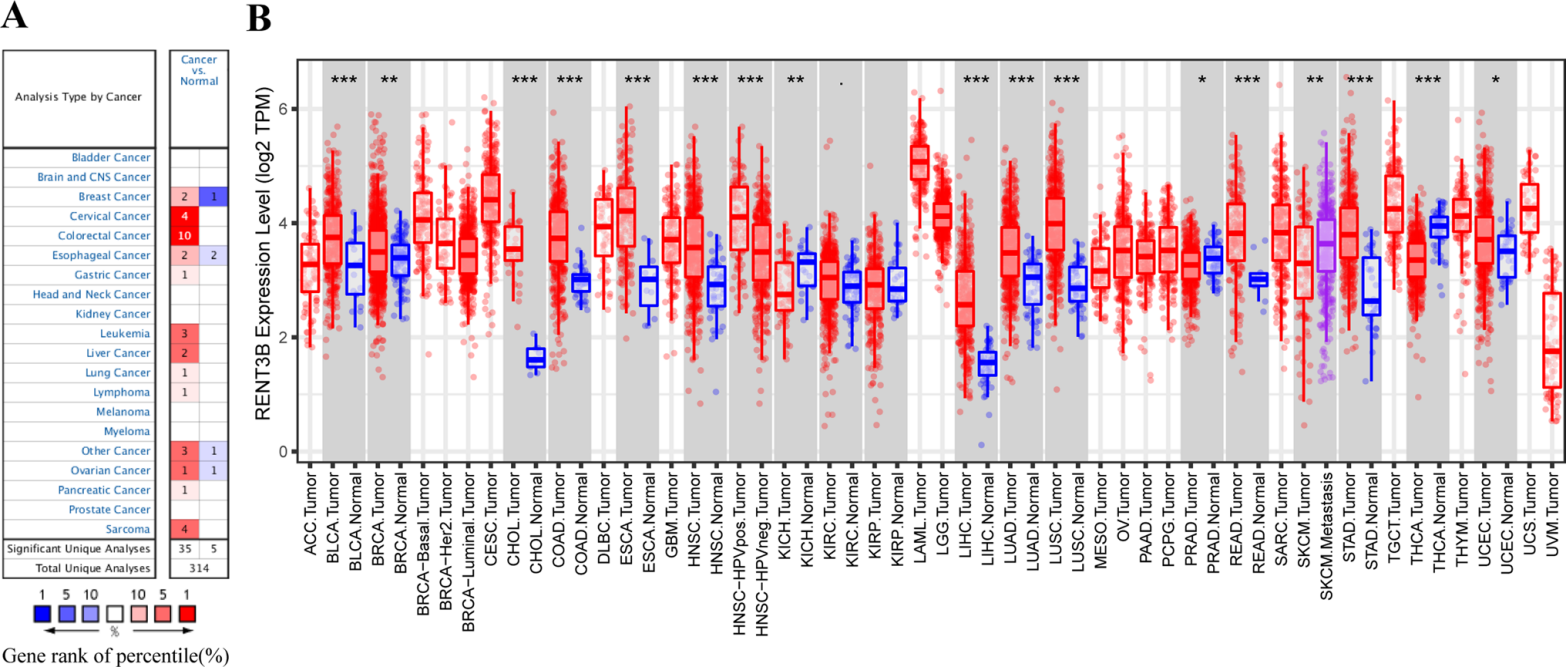
The expression levels of RENT3B in different types of cancer. **A** The expression of RENT3B in different cancers compared to normal tissues. **B** RENT3B expression levels of various tumor types in the TCGA database were determined by TIMER (*P < 0.05, **P < 0.01, ***P < 0.001)

To investigate the alteration of RENT3B expression levels in various cancers, we conducted an analysis of RENT3B mRNA expression using the ONCOMINE database. Our results revealed that compared to the control group, RENT3B expression was upregulated in Medullary Breast Carcinoma, Ductal Breast Carcinoma, Cervical Cancer, Colorectal Cancer, Esophageal Squamous Cell Carcinoma, Gastric Cancer, Leukemia, Liver Cancer, Lung Cancer, Lymphoma, Other Cancer (Vulvar Intraepithelial Neoplasia, Embryonal Carcinoma, Mixed Germ Cell Tumor), Ovarian Serous Cystadenocarcinoma, Pancreatic Cancer, and Sarcoma. Conversely, RENT3B was downregulated in Invasive Breast Carcinoma, Esophageal Adenocarcinoma, Barrett’s Esophagus, Other Cancer (Yolk Sac Tumor), and Ovarian Carcinoma (Fig. 1A). The detailed changes in RENT3B expression across pan-cancer are summarized in Supplementary Table 1.

Furthermore, we analyzed the RNA-seq data of malignant tumors from TCGA in the TIMER database to gain a deeper understanding of RENT3B expression in different tumors and healthy tissues. The results of different expression patterns of *RENT3B* in pan-cancer are shown in Fig. 1B. High expression of RENT3B was observed in BRCA (Breast invasive carcinoma), CHOL (Cholangiocarcinoma), BLCA (Bladder Urothelial Carcinoma), ESCA (Esophageal carcinoma), COAD (Colon adenocarcinoma), HNSC (Head and Neck squamous cell carcinoma), HNSC-HPV positive (Head and Neck Squamous Carcinoma-HPV positive), LIHC, LUAD (Lung adenocarcinoma), STAD (Stomach adenocarcinoma), READ (Rectum adenocarcinoma), LUSC, and UCEC (Uterine Corpus Endometrial Carcinoma). Conversely, the expression of RENT3B was downregulated in KICH (Kidney Chromophobe), PRAD (Prostate adenocarcinoma), and THCA (Thyroid carcinoma). However, there were no significant changes detected in KIRC and KIRP (Kidney renal papillary cell carcinoma). Additionally, we observed variations in RENT3B expression levels among different subtypes of esophageal carcinoma, breast cancer, and ovarian cancer.

### The prognosis of RENT3B in different cancers

**Fig. 2:**
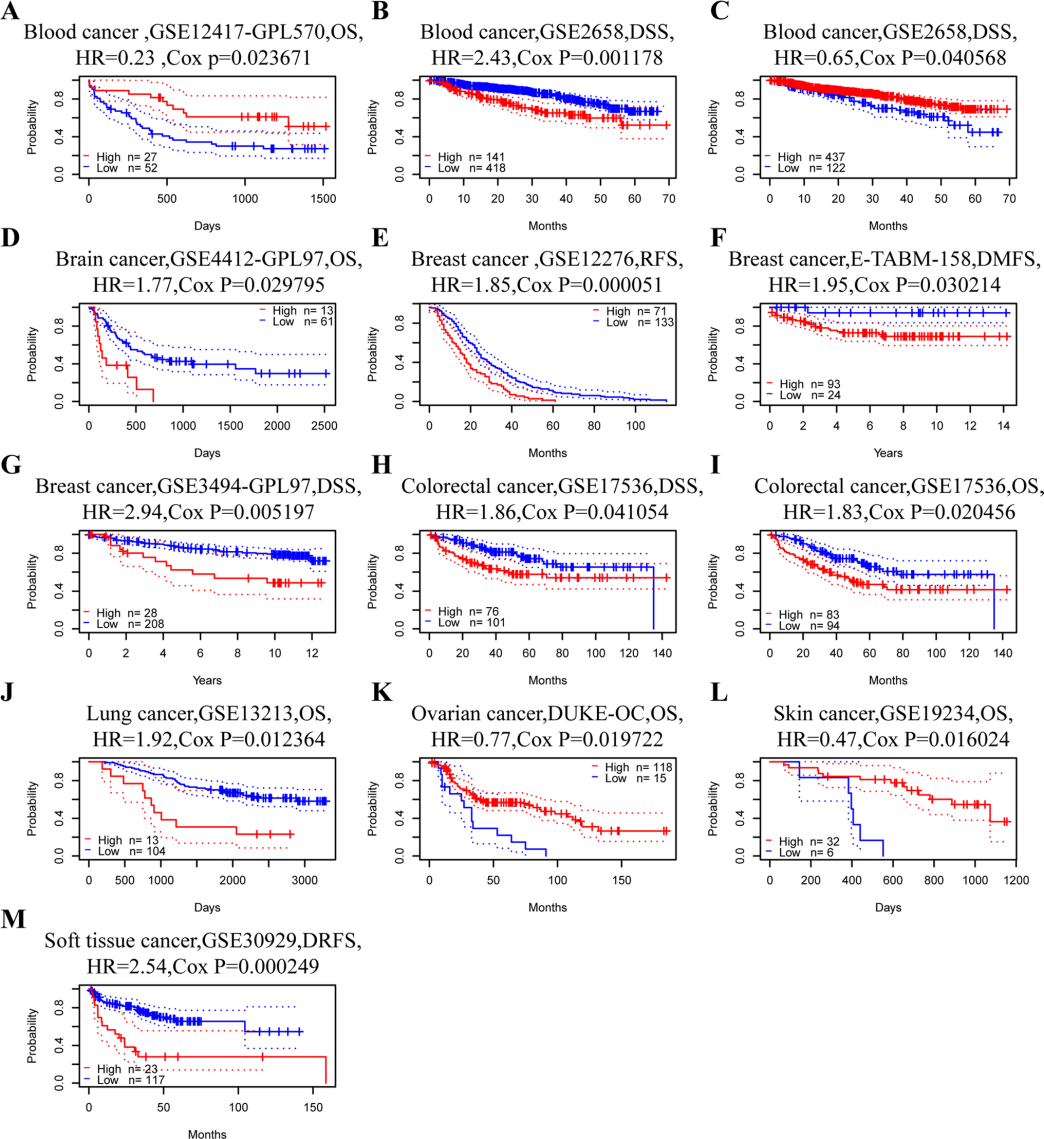
Kaplan–Meier survival curves comparing high and low expression of RENT3B in different cancer types in PrognoScan. **A** OS (n = 77) in blood tumor cohort GSE12417-GPL570. **B**, **C** DSS (n = 559) and DSS (n = 559) in blood tumor cohort GSE2658, and there is different probes of RENT3B. **D** OS (n = 74) in brain cancer cohort GSE441-GPL97. **E** RFS (n = 204) in breast cancer cohort GSE12276. **F** DMFS (n = 117) in breast cancer cohort E-TABM-158. **G** DSS (n = 236) in breast cancer cohort GSE3494-GPL97. **H, I** DSS (n = 177) and OS (n = 177) in colorectal cancer cohort GSE17536. **J** OS (n = 117) in lung cancer cohort GSE13213. **K** OS (n = 133) in ovarian cancer cohort DUKE-OS. **L** OS (n = 38) in skin cancer cohort GSE19234. **M** DRFS (n = 140) in soft tissue tumor cohort GSE30929. OS, overall survival; DSS, disease-specific survival; RFS, relapse-free survival; DMFS, distant metastasis-free survival; DRFS, distant recurrence free survival

We would like to further explore the correlation between RENT3B expression and prognosis in various tumor types to determine whether this gene acts as a promoter or inhibitor in different cancers. Initially, we assessed the correlation between RENT3B expression and prognosis in each cancer type using PrognoScan. The prognostic factors included overall survival (OS), disease-specific survival (DSS), recurrence-free survival (RFS), distant metastasis-free survival (DMFS), and disease-free survival (DRFS). The results indicated that RENT3B expression was significantly associated with eight types of cancers: blood tumor, brain cancer, breast cancer, colorectal cancer, lung cancer, ovarian cancer, skin cancer, and soft tissue tumor (Fig. 2). Among these, RENT3B had a poor prognosis in six cancer types: blood tumor (OS: HR = 2.43), brain cancer (OS: HR = 1.77), breast cancer (RFS: HR = 1.85; DSS: HR = 2.94; DMFS: HR = 1.95), colorectal cancer (OS: HR = 1.83; DSS: HR = 1.86), lung cancer (OS: HR = 1.92), and soft tissue tumor (DRFS: HR = 2.54). However, RENT3B had a favorable prognosis in three cancer types: blood tumor (DSS: HR = 0.323), ovarian cancer (OS: HR = 0.77), and skin cancer (OS: HR = 0.47). These results are summarized in Table 1. Moreover, in the GSE2658 dataset of blood tumor, two corresponding probes of the RENT3B gene showed diametrically opposite prognoses.

**Table 1:** Correlations between RENT3B expressions and prognoses in different cancers in PrognoScan database

Cancer type	Dataset	Subtype	Endpoint	N	COX p-value	HR [95% CI-low CI-up]
Blood cancer	GSE2658	Multiple myeloma	Disease Specific Survival	559	1.1776E-03	2.43 [1.42–4.16]
	GSE12417-GPL570	AML	Overall Survival	79	2.3671E-02	0.23 [0.06–0.82]
	GSE2658	Multiple myeloma	Disease Specific Survival	559	4.0568E-02	0.65 [0.43–0.98]
Brain cancer	GSE4412-GPL97	Glioma	Overall Survival	74	2.9795E-02	1.77 [1.06–2.95]
Breast cancer	GSE12276		Relapse Free Survival	204	5.1252E-05	1.85 [1.37–2.49]
	GSE3494-GPL97		Disease Specific Survival	236	5.1971E-03	2.94 [1.38–6.26]
	E-TABM-158		Distant Metastasis Free Survival	117	3.0214E-02	1.95 [1.07–3.55]
Colorectal cancer	GSE17536		Overall Survival	177	2.0456E-02	1.83 [1.10–3.06]
	GSE17536		Disease Specific Survival	177	4.1054E-02	1.86 [1.03–3.37]
Lung cancer	GSE13213	Adenocarcinoma	Overall Survival	117	1.2364E-02	1.92 [1.15–3.21]
Ovarian cancer	DUKE-OC		Overall Survival	133	1.9722E-02	0.77 [0.61–0.96]
Skin cancer	GSE19234	Melanoma	Overall Survival	38	1.6024E-02	0.47 [0.26–0.87]
Soft tissue cancer	GSE30929	Liposarcoma	Distant Recurrence Free Survival	140	2.4945E-04	2.54 [1.54–4.18]

**Fig. 3:**
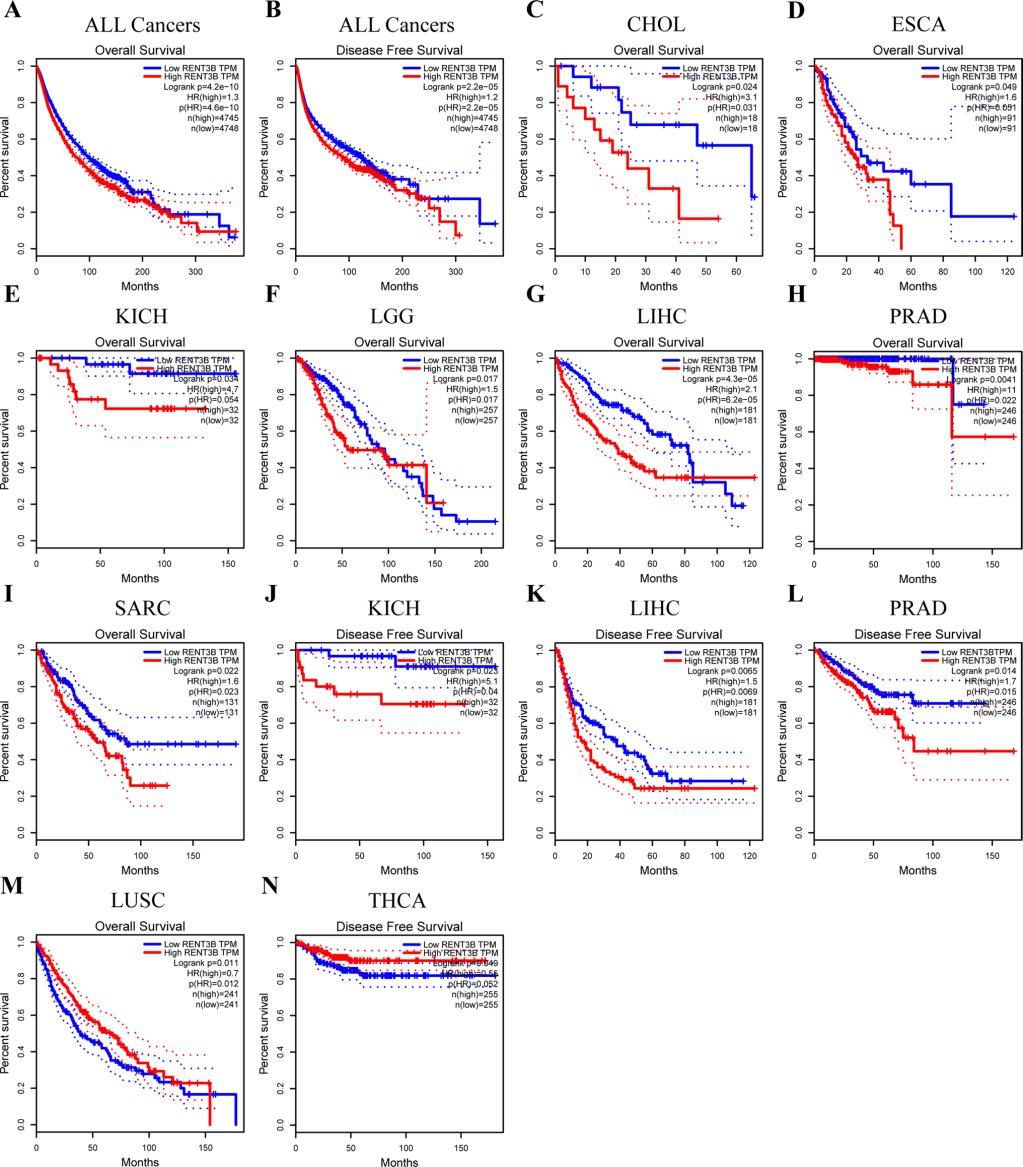
Correlation of RENT3B expression with prognostic values in cancers. Overall survival or disease-free survival of (**A, B**) all cancer types (C)CHOL, Cholangio carcinoma (**D**) ESCA, Esophageal carcinoma (**E, J**) KICH, Kidney Chromophobe (**F**) LGG, Brain Lower Grade Glioma (**G, K**) LIHC, Liver hepatocellular carcinoma (**H, L**) PRAD, Prostate adenocarcinoma (**I**) SARC, Sarcoma (**M**) LUSC, Lung squamous cell carcinoma (**N**) THCA, Thyroid carcinoma in GEPIA

Subsequently, we used data from 33 cancer types in the TCGA database through GEPIA to investigate the relationship between RENT3B expression and OS and DFS separately. Overall, RENT3B was found to be an indicator of poor prognosis in most cancers (RENT3B, OS: n = 9493, HR = 1.3, log-rank p = 4.6e-10; DFS: n = 9502, HR = 1.2, log-rank p = 2.2e-05) (Fig. 3A, B). Specifically, high expression of RENT3B was associated with worse overall survival in CHOL, ESCA, KICH, LGG, LIHC, PRAD, and SARC, and worse disease-free survival in KICH, LIHC, and PRAD (Fig. 3C–L). On the contrary, high RENT3B expression indicated better overall survival in LUSC and better disease-free survival in THCA (Fig. 3M, N).

**Fig. 4:**
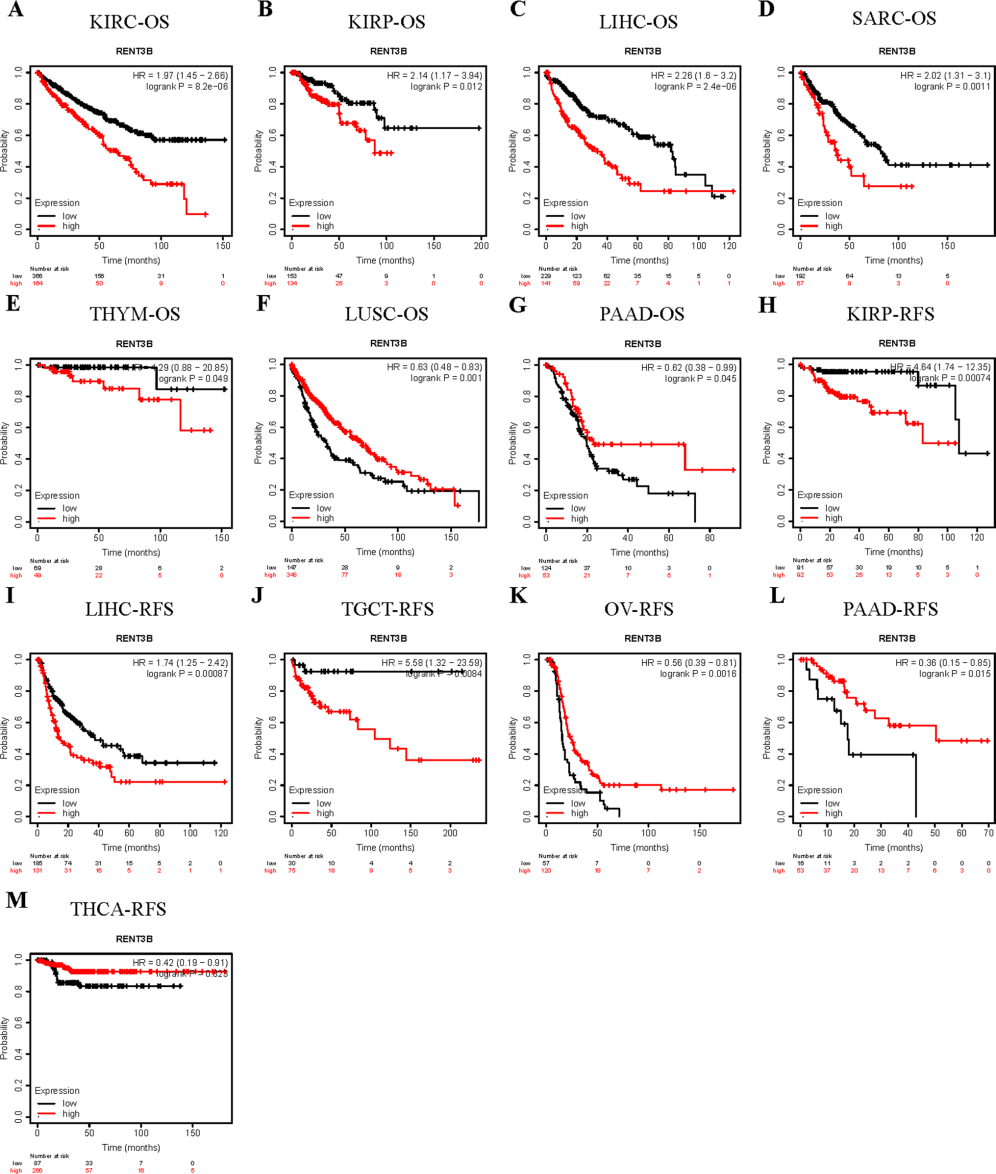
Kaplan–Meier survival curves comparing the high and low expression levels of RENT3B in various cancers. OS or RFS of (**A**) KIRC, Kidney renal clear cell carcinoma (**B, H**) KIRP, Kidney renal papillary cell carcinoma (**C, I**) LIHC, Liver hepatocellular carcinoma (**D**) SARC, Sarcoma (**E**) THYM, Thymoma (**F**) LUSC, Lung squamous cell carcinoma (**G, L**) PAAD, Pancreatic adenocarcinoma (**J**) TGCT, Testicular Germ Cell Tumors (**K**) OV, Ovarian serous cystadenocarcinoma (**M**) THCA, Thyroid carcinoma in Kaplan Meier plotter database. Red curve indicates patients with high expression of RENT3B. OS, overall survival; RFS, relapse-free survival

To further evaluate the survival association of RENT3B in various cancers, we utilized the Kaplan–Meier Plotter, which integrates data from the TCGA, EGA, and GEO databases. This analysis included OS and RFS in 21 cancer types. High expression of RENT3B was significantly associated with adverse overall survival in KIRC, KIRP, LIHC, SARC, THYM, and adverse recurrence-free survival in KIRP, LIHC, and TGCT (Fig. 4A–E, H–J). Conversely, high RENT3B expression indicated better overall survival in LUSC and PAAD, as well as better recurrence-free survival in OV, PAAD, and THCA (Fig. 4F, G, K–M). Notably, RENT3B exhibited no significant correlation with other cancer types.

Then, we have incorporated numerous prognostic indicators and conducted corresponding univariate and multivariate Cox analyses. For Liver Hepatocellular Carcinoma, the added prognostic indicators included age, Child–Pugh stage, Gender, histologic grade, liver cirrhosis, TNM stage, tumor stage, and vascular invasion. In the univariate Cox analysis, M stage, T stage, tumor stage, and RENT3B were identified as independent adverse prognostic factors (all Hazard Ratios [HRs] exceeded 1). Moreover, in the multivariate Cox analysis that combined these four factors, the HR of RENT3B continued to exceed 1 (Table 2). These dual analysis results collectively suggest a positive correlation between an elevated expression of RENT3B and an increased mortality risk among patients. In other words, as RENT3B expression rises, patient mortality risk proportionately increases. The prognostic indicators incorporated for LUSC include Age, EGFR mutation, EML4-ALK translocation, Gender, TNM stage, new tumor event, and Tumor stage. Univariate Cox analysis results indicate that M stage, T stage, new tumor event, and Tumor stage act as independent adverse prognostic factors, each with HRs exceeding 1. Interestingly, the HR of RENT3B is below 1 (HR = 0.769), suggesting that high RENT3B expression correlates with a positive prognosis. This is further confirmed by multivariate Cox analysis, which consistently identifies high RENT3B expression as a favorable prognostic factor (HR = 0.606) (Table 3). To conclude, an increase in RENT3B expression is associated with a higher risk of mortality in patients diagnosed with LIHC. However, this correlation is inverse for patients with LUSC as the risk of mortality decreases with an increase in RENT3B expression. This disparity further substantiates that RENT3B can serve as a valuable prognostic indicator, exhibiting varying outcomes in LIHC and LUSC.

**Table 2:** Univariate and Multivariate analyses of prognostic parameters in LIHC

Prognostic parameters	Univariate analysis			Multivariate analysis		
	HR	95%CI	*p* value	HR	95%CI	*p* value
Age (≥ 60 vs. < 60)	0.812	0.572–1.151	0.24201044	NA	NA	NA
Child–Pugh stage (A vs. B + C)	1.616	0.797–3.275	0.18293092	NA	NA	NA
Gender (Male vs. Female)	0.834	0.585–1.19	0.31769585	NA	NA	NA
Histologic grade (G4 + G3 vs. G2 + G1)	1.144	0.798–1.64	0.46481719	NA	NA	NA
Liver cirrhosis (Yes vs. No)	0.747	0.453–1.234	0.25460995	NA	NA	NA
M stage (M1 vs. M0)	3.889	1.223–12.367	0.02137948	2.633	0.782–8.8672	0.118052452
N stage (N1 + N2 + N3 vs. N0)	1.956	0.479–7.98	0.34990276	NA	NA	NA
T stage (T3 + T4 vs. T2 + T1)	2.549	1.79–3.631	2.17E-07	1.904	0.258–14.0615	0.527981969
Tumor stage (IV + III vs. II + I)	2.446	1.685–3.55	2.54E-06	1.362	0.185–10.0205	0.761271243
Vascular invasion (Yes vs. No)	1.323	0.871–2.008	0.18955356	NA	NA	NA
RENT3B (High vs. Low)	**1.74**	1.226–2.468	**0.00191921**	**1.573**	1.012–2.4445	**0.044056531**

**Table 3:** Univariate and Multivariate Analyses of prognostic parameters in LUSC

Prognostic parameters	Univariate analysis			Multivariate analysis		
	HR	95% CI	*p* value	HR	95% CI	*p* value
Age (≥ 60 vs. < 60)	0.791	0.534–1.172	0.242069843	NA	NA	NA
EGFR mutation (Yes vs. No)	1.078	0.526–2.211	0.837079897	NA	NA	NA
EML4 ALK translocation (Yes vs. No)	0.711	0.175–2.885	0.632672156	NA	NA	NA
Gender (Male vs. Female)	1.184	0.859–1.632	0.303570879	NA	NA	NA
M stage (M1 vs. M0)	3.107	1.269–7.605	0.013068581	3.128	0.911–10.7435	0.07001048
N stage (N1 + N2 + N3 vs. N0)	1.149	0.868–1.521	0.331827504	NA	NA	NA
T stage (T3 + T4 vs. T2 + T1)	1.705	1.233–2.357	0.001247179	1.567	0.912–2.6919	0.1036299
New tumor event (Yes vs. No)	2.593	1.866–3.605	1.42E-08	2.561	1.78–3.6826	3.95E-07
Tumor stage (IV + III vs. II + I)	1.566	1.136–2.159	0.006172146	0.938	0.519–1.6977	0.83366567
RENT3B (High vs. Low)	**0.769**	0.585–1.01	**0.04880452**	**0.606**	0.421–0.8739	**0.00729772**

Based on these findings, RENT3B generally served as a poor prognostic factor. In LIHC, the overall survival, disease-free survival, and recurrence-free survival associated with RENT3B were consistent with the overall results. However, we observed the opposite effect of RENT3B in LUSC, where it acted as a tumor suppressor. This discrepancy piqued our interest, leading us to select the RENT3B gene for further investigation in LIHC and LUSC.

### The relationship between RENT3B expression and immune infiltration

**Fig. 5:**
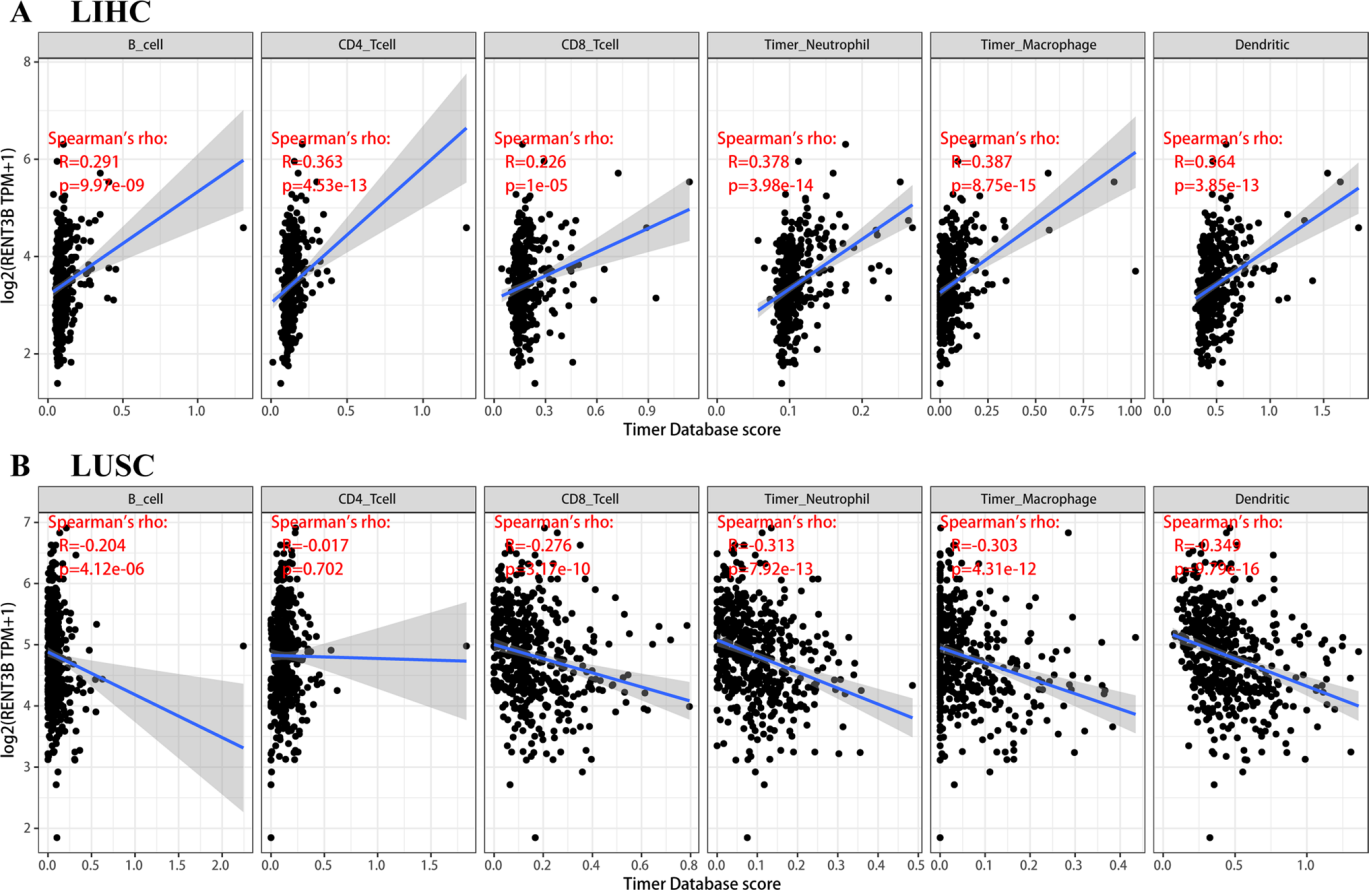
Correlation of RENT3B expression with immune infiltration in LIHC and LUSC in TIMER. **A** RENT3B expressions were positively correlated with infiltrating levels of B cell, CD4^+^T cell, CD8^+^T cell, neutrophil, macrophage, and dendritic. **B** RENT3B expressions were negatively correlated with infiltrating levels of B cell, CD8^+^T cell, neutrophil, macrophage, and dendritic

Some previous studies have demonstrated the impact of immune cells in the tumor microenvironment on patients’ prognosis. Therefore, we aimed to investigate the potential association between RENT3B expression and immune infiltration in LIHC and LUSC. To determine the interrelationship between RENT3B expression and immune cells in the tumor microenvironment, we utilized the TIMER database. In LIHC, we observed an inverse correlation between RENT3B expression and B cell (R = 0.291, p = 9.97e-09), CD4 + T cell (R = 0.363, p = 4.53e-13), CD8 + T cell (R = 0.226, p = 1e-05), neutrophil (R = 0.378, p = 3.98e-14), macrophage (R = 0.387, p = 8.75e-15), and dendritic cell (R = 0.364, p = 3.85e-13) infiltration (Fig. 5A). Conversely, in LUSC, RENT3B showed a positive correlation with B cell (R = − 0.204, p = 4.12e-06), CD8 + T cell (R = − 0.276, p = 3.17e-10), neutrophil (R = − 0.313, p = 7.92e-13), macrophage (R = − 0.303, p = 4.31e-12), and dendritic cell (R = − 0.349, p = 9.79e-16) infiltration (Fig. 5B). These findings clearly indicate a direct relationship between RENT3B expression and immune infiltration in LIHC, whereas a negative relationship was observed in LUSC.

Based on our comprehensive analysis, it is evident that RENT3B influences patient prognosis by modulating immune infiltration in both LIHC and LUSC. This result provides a possible explanation for the unfavorable prognostic factor of RENT3B in LIHC and its favorable prognostic factor in LUSC.

### Correlation between RENT3B and immune cell marker

We planned to investigate further the correlation between the expression of RENT3B and markers of various immune cells in the TIMER database. We selected 45 immune cell markers, involving 19 types of immune cells: B cell, CD8^+^ T cell, Thf, Th1, Th2, Th9, Th17, Th22, Treg, exhausted T cells, Macrophage, M1/M2 macrophage, Monocyte, TAM, NK, Neutrophil, DC.

RENT3B showed a significant correlation with almost all immune cell markers in LIHC, except NOS2 and FUT4. Among them, RENT3B was negatively correlated only with ARG1 in M2 and CD14 in Monocyte, and positively correlated with the rest. Importantly, these correlation results remained consistent even after considering tumor purity. Notably, there were strong positive correlation coefficients observed for CD38 (Cor = 0.32830733) in B cells, ICOS (Cor = 0.35149583) and BCL6 (Cor = 0.38061806) in Thf, IL12RB2 (Cor = 0.3053123) and IL27RA (Cor = 0.43571992) in Th1, CCR3 (Cor = 0.42179223) and GATA3 (Cor = 0.30572785) in Th2, TGFBR2 (Cor = 0.318355), IRF4 (Cor = 0.34292587), and SPI1 (Cor = 0.43936172) in Th9, IL21R (Cor = 0.41680877) and STAT3 (Cor = 0.40314791) in Th17, CCR10 (Cor = 0.36084895) in Th22, CCR8 (Cor = 0.49796427) and IL2RA (Cor = 0.42935477) in Treg, PDCD1 (Cor = 0.30167985) and CTLA4 (Cor = 0.35324917) in T cell exhaustion, CD68 (Cor = 0.3345455) and ITGAM (Cor = 0.41813729) in Macrophage, CD80 (Cor = 0.44930451) and CD86 (Cor = 0.42657434) in TAM, FCGR3A (Cor = 0.42997605) in Monocyte, and FUT4 (Cor = 0.4565848) in Neutrophil after considering tumor purity. These findings indicate that RENT3B is associated with these 18 immune cells in LIHC. In LUSC, RENT3B is correlated with 36 immune markers. However, these correlation results slightly decreased when considering tumor purity, and RENT3B showed significant correlations with only 19 immune markers. Among these 19 markers, 13 exhibited negative correlation coefficients, which corresponded to Thf, Th9, Th22, Treg, Macrophage, M2, TAM, Monocyte, and DC (Table 4).

**Table 4:** Correlation analysis between RENT3B and immune cell type markers in TIMER database

Cell type	Gene marker	LIHC				LUSC				
		None		Purity		None			Purity	
		Cor	*P*	Cor	*P*	Cor	*P*		Cor	*P*
B Cell	CD19	0.258727	***	0.28698734	***	− 0.09503	*		0.055153	
	MS4A1	0.116874	*	0.15932186	**	− 0.0757			0.07086	
	CD38	0.263327	***	0.32830733	***	− 0.13593	**		− 0.06288	
CD8 + T Cell	CD8A	0.200286	***	0.2565562	***	− 0.15527	***		− 0.08005	
	CD8B	0.128614	*	0.17703867	***	− 0.09896	*		− 0.06313	
Thf	CXCR5	0.188115	***	0.2434024	***	− 0.07744			0.081372	
	ICOS	0.264942	***	0.35149583	***	− 0.21213	***		− 0.10616	*
	BCL6	0.37643	***	0.38061806	***	0.292219	***		0.269096	***
Th1	IL12RB2	0.307446	***	0.3053123	***	− 0.02127			0.023393	
	IL27RA	0.385243	***	0.43571992	***	0.022366			0.063116	
	TBX21	0.143725	**	0.18911557	***	− 0.05777			0.053801	
Th2	CCR3	0.370275	***	0.42179223	***	− 0.16355	***		− 0.09407	*
	STAT6	0.327995	***	0.29391503	***	0.200499	***		0.217357	***
	GATA3	0.214019	***	0.30572785	***	− 0.09998	*		− 0.04033	
Th9	TGFBR2	0.303021	***	0.318355	***	− 0.24689	***		− 0.14855	**
	IRF4	0.266861	***	0.34292587	***	− 0.08996	*		0.05626	
	SPI1	0.333282	***	0.43936172	***	− 0.21649	***		− 0.09296	*
Th17	IL21R	0.324948	***	0.41680877	***	− 0.14693	***		− 0.02528	
	IL23R	0.272172	***	0.27867336	***	0.002882			0.081797	
	STAT3	0.385856	***	0.40314791	***	0.121256	**		0.151405	***
Th22	CCR10	0.339312	***	0.36084895	***	− 0.14423	**		− 0.08622	
	AHR	0.277777	***	0.28128734	***	− 0.1506	***		− 0.13621	**
Treg	FOXP3	0.234103	***	0.24892093	***	− 0.09796	*		0.026437	
	CCR8	0.429812	***	0.49796427	***	− 0.12767	**		− 0.02861	
	IL2RA	0.344081	***	0.42935477	***	− 0.21319	***		− 0.12874	**
T cell exhaustion	PDCD1	0.241481	***	0.30167985	***	− 0.08818	*		0.020951	
	CTLA4	0.274083	***	0.35324917	***	− 0.09167	*		0.033182	
Macrophage	CD68	0.269446	***	0.3345455	***	− 0.27607	***		− 0.18944	***
	ITGAM	0.361413	***	0.41813729	***	− 0.08824	*		0.028923	
M1	NOS2	0.091313		0.08692134		0.214358	***		0.20685	***
	ROS1	0.219953	***	0.20938038	***	− 0.14987	***		− 0.07116	
M2	ARG1	− 0.19119	***	− 0.19992131	***	0.040843			0.038302	
	MRC1	0.108917	*	0.12599962	*	− 0.23425	***		− 0.14353	**
TAM	HLA-G	0.197759	***	0.19154881	***	− 0.05982			0.010555	
	CD80	0.376909	***	0.44930451	***	− 0.24007	***		− 0.15774	***
	CD86	0.329481	***	0.42657434	***	− 0.27601	***		− 0.1816	***
Monocyte	CD14	− 0.29228	***	− 0.29594981	***	− 0.32502	***		− 0.23014	***
	FCGR3A	0.370164	***	0.42997605	***	− 0.29009	***		− 0.21327	***
NK	XCL1	0.255527	***	0.28733954	***	0.227955	***		0.204352	***
	KIR3DL1	0.131863	*	0.13662685	*	− 0.07197			− 0.02786	
	CD7	0.220835	***	0.26815455	***	− 0.11723	**		− 0.00195	
Neutrophil	FUT4	0.435389		0.4565848	***	0.157745	***		0.214515	***
	MPO	0.121979	*	0.14561821	**	− 0.0982	*		− 0.02858	
DC	CD1C	0.134203	**	0.17869505	***	− 0.28461	***		− 0.16808	***
	THBD	0.110285	*	0.15847987	**	− 0.08849	*		− 0.07121	

*LIHC* liver hepatocellular carcinoma, *LUSC* lung squamous cell carcinoma, *Tfh* follicular helper T cell, *Th* T helper cell, *Treg* Regulatory T cell, *TAM* tumor-associated- macrophage, *NK* natural killer cell, *DC* dendritic cell, *None* correlation without adjustment, *Purity* correlation adjusted for tumor purity, *Cor* r value of Spearman’s correlation. *P < 0.01; **P < 0.001; ***P < 0.0001

Overall, these findings suggest that the expression of RENT3B is associated with immune infiltration in both LIHC and LUSC, with different correlations observed in each tumor type. Combined with previous prognosis results, it can be inferred that the impact of RENT3B expression on prognosis is most likely due to immune infiltration in these two tumors. Furthermore, these results demonstrate tissue heterogeneity of RENT3B in LIHC and LUSC, as it influences immune infiltration in different tumor tissues, leading to varying survival outcomes.

### Survival analysis of RENT3B expressions in pan-cancer based on immune cells

**Fig. 6:**
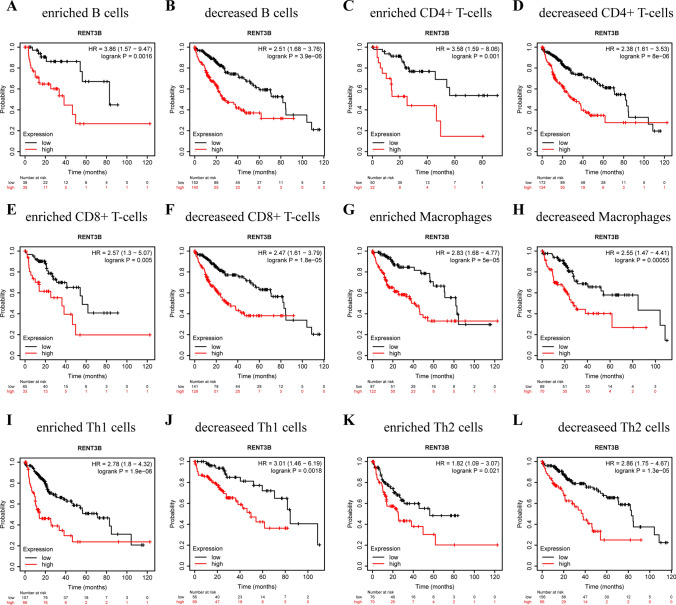
Comparison of overall survival curves of the high and low expression of RENT3B in LIHC based on immune cells subgroups. Correlations between RENT3B of different immune cells subgroup and prognoses in (**A**–**L**) LIHC in Kaplan Meier plotter database. Red curve indicates patients with high expression of RENT3B

After discovering the correlation between RENT3B expression and immune infiltration, we employed the Kaplan–Meier Plotter to divide LIHC and LUSC samples into enriched and decreased subgroups based on immune cell subtypes. Our objective was to investigate whether specific types of immune cells have an impact on tumor prognosis. Building upon this, we further investigated the relationship between RENT3B expression and survival outcomes. As expected, our results revealed that HCC patients with high expression of RENT3B exhibited a poorer prognosis in the enriched and decreased cohorts of B cells, CD4^+^ T cells, CD8^+^ T cells, Macrophages, Th1 cells, and Th2 cells (Fig. 6). Conversely, in the enriched and decreased cohorts of B cells, CD4^+^ T cells, CD8^+^ T cells, Macrophages, Th1 cells, and Th2 cells in LUSC, high RENT3B expression was associated with a more favorable prognosis [Supplementary Fig. 1]. This finding aligns with our previous survival analysis that did not differentiate among immune cell subgroups. By analyzing these results, we can conclude that among the six types of immune cells examined, only a particular type did not impact the survival rates of LIHC and LUSC patients.

### The high expression of RENT3B is not due to promoter methylation in LIHC and LUSC

Generally speaking, the transcriptional activity of a gene is inversely proportional to the methylation of CpG sites in the gene promoter. Therefore, upon discovering the relationship between the prognosis of RENT3B and immune infiltration, we aimed to investigate whether the upregulation of RENT3B expression is caused by promoter methylation in LIHC and LUSC.

To analyze DNA methylation, the DNMIVD database utilizes high-throughput microarray data from TCGA and GEO. According to the results obtained from DNMIVD, there was only a marginal difference in the promoter methylation of RENT3B between normal and tumor samples in LIHC, and this difference was not statistically significant (p > 0.05). Similarly, no statistical difference (p > 0.05) in promoter methylation was observed in LUSC (Supplementary Fig. 2). These findings indicate that the high expression of RENT3B is unrelated to promoter methylation in both LIHC and LUSC.

## Discussion

In this study, we aimed to investigate the role of RENT3B in pan-cancers, specifically its expression, prognosis, and, most importantly, its association with immune infiltration. Understanding the tumor microenvironment, particularly immune cell infiltration, is crucial for elucidating the mechanisms underlying tumor development. We identified a significant relationship between RENT3B and immune cell infiltration. The interaction patterns varied between LIHC and LUSC. However, it is important to emphasize that our current research design does not establish a causal relationship between RENT3B and immune infiltration. Therefore, this study focuses on investigating the potential of RENT3B as a prognostic marker for pan-cancers from an immuno-oncology perspective. These findings may have implications for future mechanistic research and contribute to the advancement of immunotherapy.

Despite variations in data collection methods among different databases, we were able to determine distinct prognostic values of RENT3B in LIHC and LUSC by combining data from multiple sources. Therefore, we found the protein expression levels of RENT3B in LIHC and LUSC on The Human Protein Atlas database. The Human Protein Atlas database utilizes immunohistochemistry to visualize protein abundance differences. As shown in the figure, RENT3B is predominantly localized in the cytoplasm. In LIHC, the protein content of RENT3B in the tumor is significantly higher than in normal liver tissue. The same result applies to LUSC (Supplementary Fig. 3).

To evaluate patient prognosis, we conducted prognostic analyses of RENT3B using the GEPIA and Kaplan–Meier Plotter platforms. The results indicated that high expression of RENT3B was associated with an unfavorable prognosis in LIHC, whereas high RENT3B expression correlated with a favorable prognosis in LUSC. Subsequently, we employed both univariate and multivariate Cox regression analyses to assess the distinct prognostic implications of RENT3B in LIHC and LUSC (Tables 2 and 3). Collectively, these findings suggest that RENT3B may serve as a prognostic biomarker across various cancer types.

Another significant finding of this study is the correlation between RENT3B expression and tumor immune infiltration, particularly in LIHC and LUSC. Our results demonstrate that RENT3B expression closely correlates with the infiltration levels of various immune cell types. In LIHC, the correlation coefficient between RENT3B expression and immune infiltration is positive, whereas in LUSC, it is negative. Regarding the association between RENT3B expression and immune cell markers, our results reveal a positive correlation between RENT3B and nearly all immune cell markers in LIHC. However, the correlation pattern differs somewhat in LUSC. Notably, the associations between RENT3B and certain immune cell markers do not precisely follow the overall trend observed, indicating a specific interaction between RENT3B expression and particular immune cell subtypes. In conclusion, our study highlights the significant role of RENT3B in regulating immune infiltrating cells in cancer, which may ultimately influence patient prognosis.

The above results demonstrate that RENT3B is an unfavorable prognostic indicator in LIHC. Conversely, in LUSC, RENT3B was found to be a factor of good prognosis. Why do opposite prognostic outcomes occur? We have conducted further analysis to address this question. In the TCGA-LIHC and TCGA-LUSC RNA-seq data, we performed differential analysis by grouping tumor samples based on the expression level of RENT3B using the minimum *p*-value method. Subsequently, we conducted KEGG and GSEA pathway enrichment analyses on the differential results. In LIHC, we observed that high expression of RENT3B is more enriched in the “Cell cycle” and “DNA replication” pathways according to KEGG analysis. GSEA analysis showed greater enrichment in the “G2M_CHECKPOINT” and “MITOTIC_SPINDLE” pathways [Supplementary Fig. 4]. These results indicate that the expression level of RENT3B is closely and positively correlated with proliferative characteristics in LIHC. In LUSC, KEGG analysis revealed that the “Natural killer cell mediated cytotoxicity” and “PD-L1 expression and PD-1 checkpoint pathway in cancer” signaling pathway are suppressed in the high RENT3B expression group. GSEA analysis indicated that RENT3B negatively correlates with the “HALLMARK_EPITHELIAL_MESENCHYMAL_TRANSITION”, and “HALLMARK_TGF_BETA_SIGNALING” pathways (Supplementary Fig. 4). Therefore, high expression of RENT3B indicates activation of tumor immunity in LUSC, with suppression of EMT and metastatic behavior. Therefore, high RENT3B expression in LUSC is associated with a better prognosis.

Previous findings have demonstrated an association between RENT3B and immune cells in the tumor microenvironment, as well as immune cell markers. Furthermore, immune cells within the immune microenvironment not only aid tumor cells in evading destruction but also participate in tumor development [2, 3]. Taking these factors into account, along with the analysis of the prognostic results of RENT3B in LIHC and LUSC, it can be concluded that RENT3B impacts the prognosis of tumor patients through immune infiltration. This finding strengthens our conclusion. RENT3B does not interact exclusively with specific types of immune cells, but rather collaborates with various immune cells to collectively influence immune infiltration. Ultimately, this interaction may impact the survival outcome of cancer patients. However, due to database limitations and technical constraints, we were unable to explore in-depth the mechanisms underlying RENT3B’s interactions with different immune cells.

## Material and methods

### The expression of RENT3B in pan-cancer in Oncomine and TIMER

The gene expression of RENT3B was examined using various tumor datasets available on the Oncomine database (https://www.oncomine.org/resource/main.html). [12] The selection criteria included a p-value threshold of 0.01, a fold change of at least 1.5, and consideration of all genes. It is important to note that the data analyzed in this study specifically involved mRNA rather than DNA. To explore the differential expression of RENT3B or any other gene of interest between tumor and healthy tissues across all TCGA data, the DiffExp module in TIMER (https://cistrome.shinyapps.io/timer/) was employed [13]. The distribution of gene expression levels was visualized using a box plot, and the significance of differential expression was determined through statistical analysis utilizing the Wilcoxon test (*: *p*-value < 0.05; **: *p*-value < 0.01; ***: *p*-value < 0.001).

### Prognosis analysis in PrognoScan, GEPIA, Kaplan–Meier plotter

The association between the expression of RENT3B and prognosis in various cancers was investigated using PrognoScan (http://dna00.bio.kyutech.ac.jp/PrognoScan/index.html), Kaplan–Meier Plotter (https://kmplot.com/analysis/), and GEPIA (http://gepia.cancerpku.cn/). [14–17]

We analyzed the correlation between RENT3B expression and survival using all available chip data from PrognoScan, including OS (Overall Survival), DSS (Disease Specific Survival), RFS (Relapse Free Survival), DMFS (Distant Metastasis Free Survival), and DRFS (Distant Recurrence Free Survival). The screening criterion used was a cox *p*-value < 0.05.

GEPIA (Gene Expression Profiling Interactive Analysis) is an interactive online platform that contains information on 9736 tumor samples from TCGA and 8587 standard samples from TCGA and GTEx projects. Its RNA-Seq datasets are derived from the UCSC Xena project. The effects of RENT3B expression on OS and DFS (Disease Free Survival) in 33 cancers are explored in GEPIA.

Kaplan–Meier Plotter is a powerful tool that can evaluate the effect of 54,000 genes on the survival rate of 21 types of cancer. The mRNA subsystem contains RNA-seq data from 74,000 samples of 21 types of tumors. The association between RENT3B and OS and RFS in various cancers, such as LIHC and LUSC, was analyzed. We also divided the tumor samples into enriched and decreased subgroups based on the immune cell subgroup in Kaplan–Meier Plotter. The HR value, 95% confidence interval, and log-rank p-value were calculated, and the screening criterion for the log-rank *p*-value was < 0.05.

### The correlation between RENT3B and immune infiltration in TIMER

We utilized the TIMER and GEPIA databases to investigate the correlation between RENT3B and immune cells. The TIMER Web server is a comprehensive resource for systematically analyzing immune infiltration in various types of cancer. The TIMER database comprises over 10,000 samples from 39 types of cancer collected from TCGA. The TIMER algorithm Synapse estimated six types of immune infiltrating cells (B cells, CD4^+^ T cells, CD8^+^ T cells, Neutrophils, Macrophages, and Dendritic cells). The Gene module of TIMER allows exploration and visualization of the association between gene expression and the abundance of immune infiltrating cells. We analyzed the correlation between RENT3B expression and the abundance of these six immune infiltrating cells across the 39 cancer types. To correct gene expression levels, we used the log2TPM method. The correlation between RENT3B and immune infiltrating cells was displayed as a scatterplot, with RENT3B gene expression on the Y-axis and immune cell infiltration level on the X-axis. The scatterplot includes Spearman’s Rho value and the estimated statistical significance (*p*-value).

In addition to assessing immune infiltrating cells, we also investigated the relationship between gene expression and immune cell markers to identify immune cell subtypes. We selected 45 markers representing various immune cells, corresponding to a total of 19 immune cell types (B cells, CD8^+^ T cells, follicular helper T cells (Tfh), T-helper 1 (Th1) cells, T-helper 2 (Th2) cells, T-helper 9 (Th9) cells, T-helper 17 (Th17) cells, T-helper 22 (Th22) cells, Regulatory T cells (Tregs), exhausted T cells, M1 macrophages, M2 macrophages, tumor-associated macrophages, monocytes, natural killer (NK) cells, neutrophils, and dendritic cells). We adjusted the correlation coefficient based on None and Purity, and recorded the correlation coefficient and P-value between RENT3B and each type of immune cell marker.

### DNMIVD database analysis

DNMIVD (DNA Methylation Interactive Visualization Database) is a comprehensive annotation and interactive visualization database for DNA methylation profiles in various human tumors [18]. It was constructed using high-throughput microarray data from the TCGA and GEO databases (http://119.3.41.228/dnmivd/index/). By utilizing DNMIVD, researchers can explore differences in promoter region methylation between tumor and normal samples. In this study, we aimed to investigate the promoter methylation level of RENT3B in LIHC and LUSC using the DNMIVD database.

### Statistical analysis

The results with P-values generated in ONCOMINE are determined by t-test, fold change, and gene grade. The statistically significant Wilcoxon test assessed the differences in expression in TIMER’s cancer samples. Survival curves were estimated using the Kaplan–Meier method, and the log-rank test was employed to calculate hazard ratios (HR) and log-rank *p*-values in Kaplan–Meier plotter and GEPIA for comparing various survival curves. Additionally, HR and Cox-*p* values were calculated in PrognoScan using the univariate Cox regression model. Spearman’s correlation was used to determine the association between RENT3B expression and immune infiltration. Unless otherwise specified, a *p*-value of less than 0.05 was considered statistically significant.

## Limitations

Although we gathered information from multiple databases, this study still has several limitations. Firstly, most of the tumor tissue information used in this study came from microarray and sequencing data. Consequently, there may be a systemic bias introduced during the analysis of immune cell markers at the cellular level. Future research with higher resolution, such as single-cell RNA sequencing, should be pursued to overcome this challenge [21, 22]. Secondly, this study solely focused on bioinformatics analysis of RENT3B expression and patient prognosis across different databases, without conducting in vivo/in vitro experiments. Thirdly, while we observed a correlation between RENT3B expression and immune cell infiltration in tumors, we cannot establish a causal relationship in our study. Fourthly, although we discovered that RENT3B can interact with various immune cells to influence immune infiltration, we were unable to determine how exactly RENT3B affects patient survival by influencing immune infiltration due to limitations in databases and technical methods. Fifthly, we identified potential heterogeneity of RENT3B in different tumor tissues, and also found differences in the ways it affects tumor immune infiltration. However, we currently lack the technical means and databases to fully explain this heterogeneity. Sixthly, gene expression upregulation or downregulation in tumor tissues is influenced by numerous factors, such as methylation and miRNA. However, in this study, we solely focused on the effect of promoter methylation on gene expression. Prospective studies investigating RENT3B expression and immune infiltration in tumor patients may provide more precise answers regarding their relationship in the future.

## Conclusion

RENT3B can impact the prognosis of cancer and is closely associated with immune infiltration, particularly in hepatocellular carcinoma and lung squamous cell carcinoma. Therefore, RENT3B has the potential to serve as a prognostic biomarker for hepatocellular carcinoma and lung squamous cell carcinoma, which may contribute to the development of immune-based anti-tumor strategies and the improvement of cancer patients’ prognosis. Furthermore, our findings will provide a new direction for molecular biology research on RENT3B.

## Supplementary Information

Additional file 1.

Additional file 2.

Additional file 3.

Additional file 4.

Additional file 5.

## Data Availability

Publicly available datasets were analyzed in this study. This data can be found here: https://www.oncomine.org/resource/main.html; https://kmplot.com/analysis/; http://dna00.bio.kyutech.ac.jp/PrognoScan/index.html; http://gepia.cancerpku.cn/; and https://cistrome.shinyapps.io/timer/; and http://119.3.41.228/ dnmivd/index/; and https://www.proteinatlas.org/.

## Abbreviations

BLCA: Bladder urothelial carcinoma
BRCA: Breast invasive carcinoma
CHOL: Cholangiocarcinoma
COAD: Colon adenocarcinoma
DFS: Disease free survival
DMFS: Distant metastasis free survival
DNMIVD: DNA methylation interactive visualization database
DRFS: Distant recurrence free survival
DSS: Disease specific survival
ESCA: Esophageal carcinoma
GEPIA: Gene expression profiling interactive analysis
HBV: Hepatitis B virus or hepatitis
HCC: Hepatocellular carcinoma
HCV: Hepatitis C virus or hepatitis
HNSC: Head and neck squamous cell carcinoma
HNSC-HPV positive: Head and neck squamous carcinoma-HPV positive
KICH: Kidney chromophobe
KIRC: Kidney renal clear cell carcinoma
KIRP: Kidney renal papillary cell carcinoma
LGG: Brain lower grade glioma
LIHC: Liver hepatocellular carcinoma
LUAD: Lung adenocarcinoma
LUSC: Lung squamous cell carcinoma
NMD: Nonsense-mediated mRNA decay
NSCLC: Non-small cell lung cancer
OS: Overall survival
OV: Ovarian serous cystadenocarcinoma
PAAD: Pancreatic adenocarcinoma
PRAD: Prostate adenocarcinoma
READ: Rectum adenocarcinoma
RFS: Relapse free survival
SARC: Sarcoma
SCLC: Small cell carcinoma
STAD: Stomach adenocarcinoma
TACE: Transcatheter arterial chemoembolization
TAM: Tumor-associated macrophages
TGCT: Testicular germ cell tumors
THCA: Thyroid carcinoma
THYM: Thymoma
TIMER: Tumor IMmune Estimation Resource
TME: Tumor microenvironment
UCEC: Uterine corpus endometrial carcinoma
