# Supplementary figures and images for: Regulator of nonsense transcripts 3B is a prognostic biomarker and associated with immune cell infiltration in lung squamous cell and hepatocellular carcinoma
Source: Discov Oncol. 2024 Sep 27;15:479. doi: 10.1007/s12672-024-01369-3 (PMC11436519; doi:10.1007/s12672-024-01369-3)

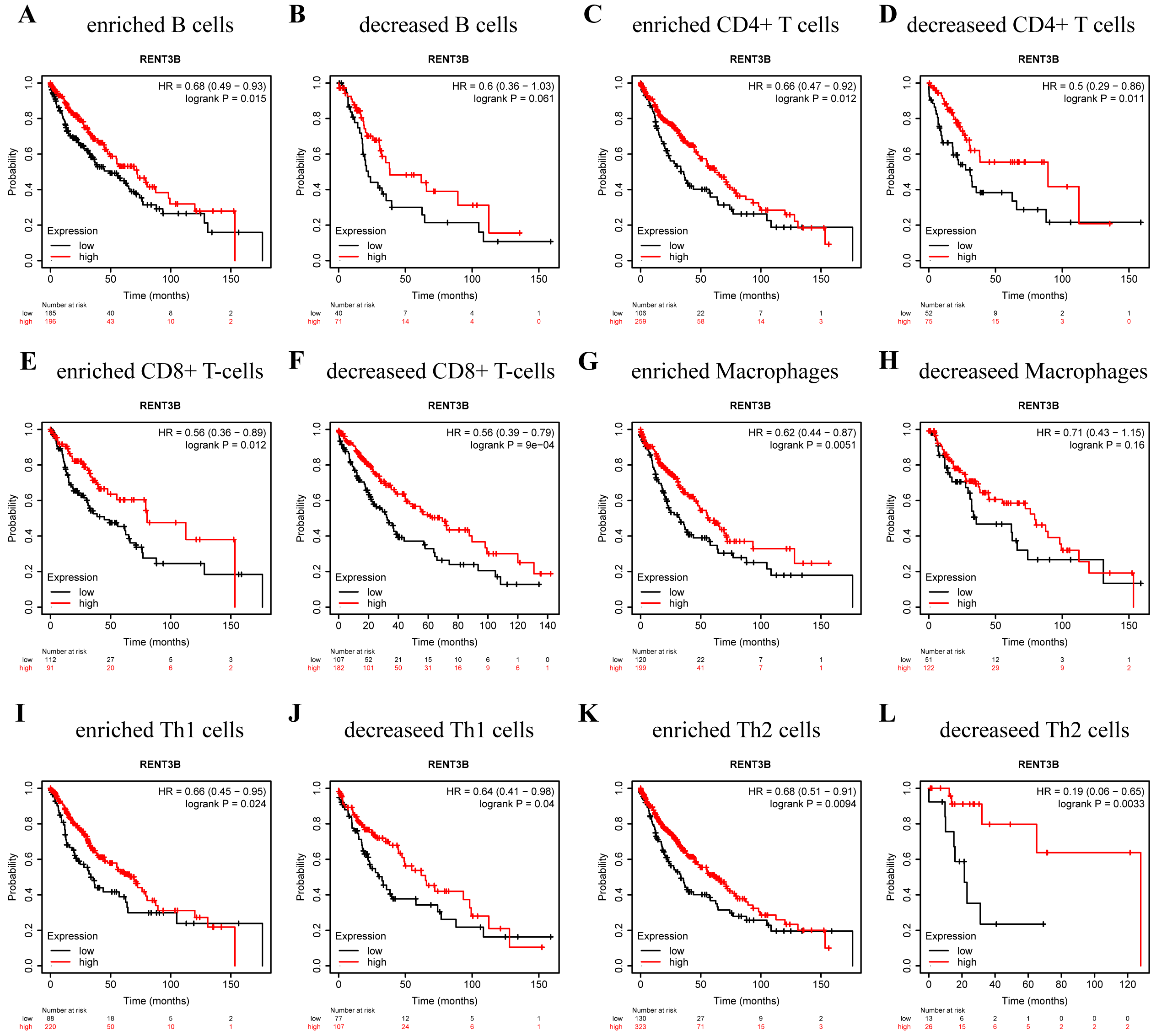

Supplement: Supplementary file 1 — Additional file 1. [file 12672_2024_1369_MOESM1_ESM.tif]

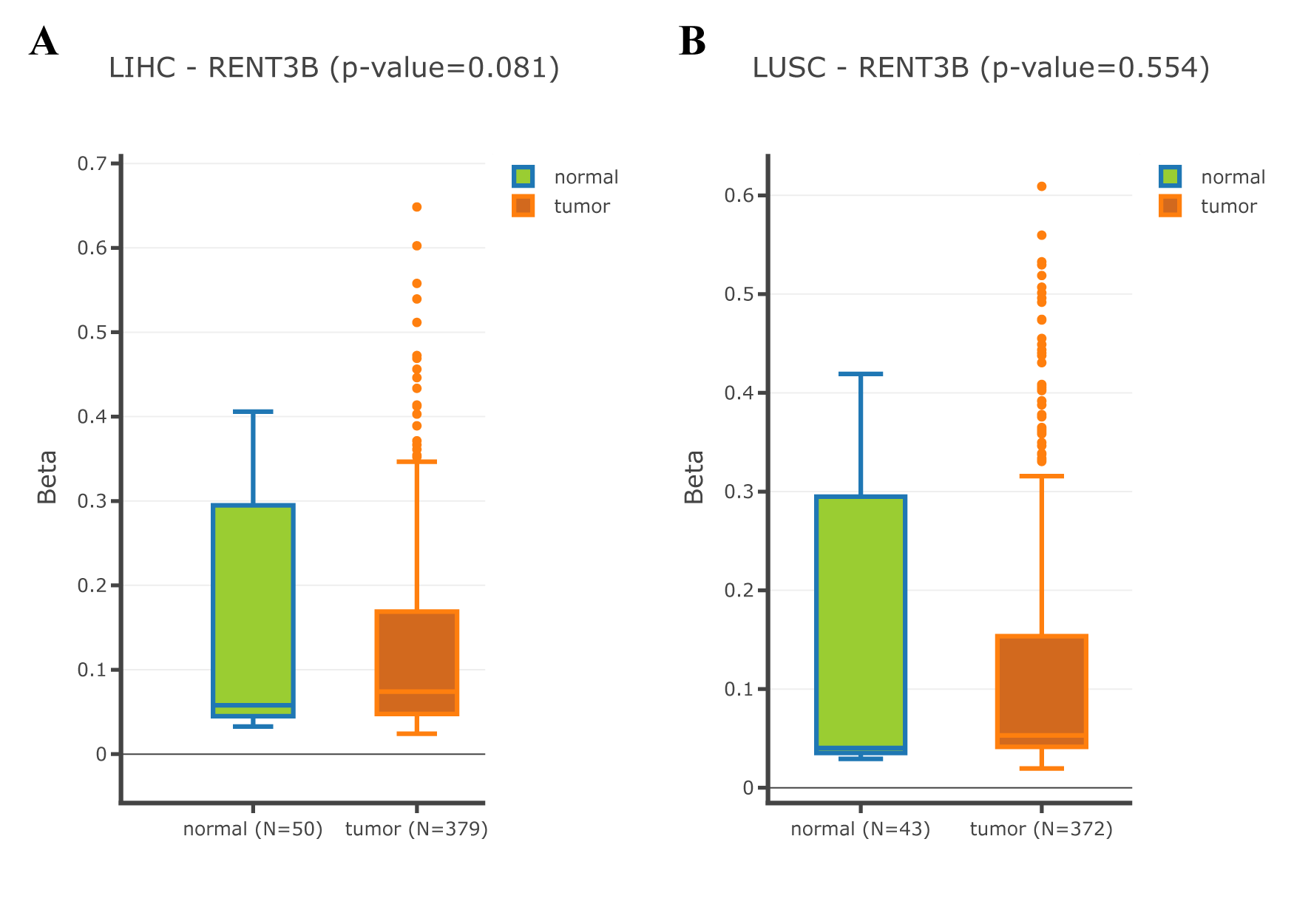

Supplement: Supplementary file 2 — Additional file 2. [file 12672_2024_1369_MOESM2_ESM.tif]

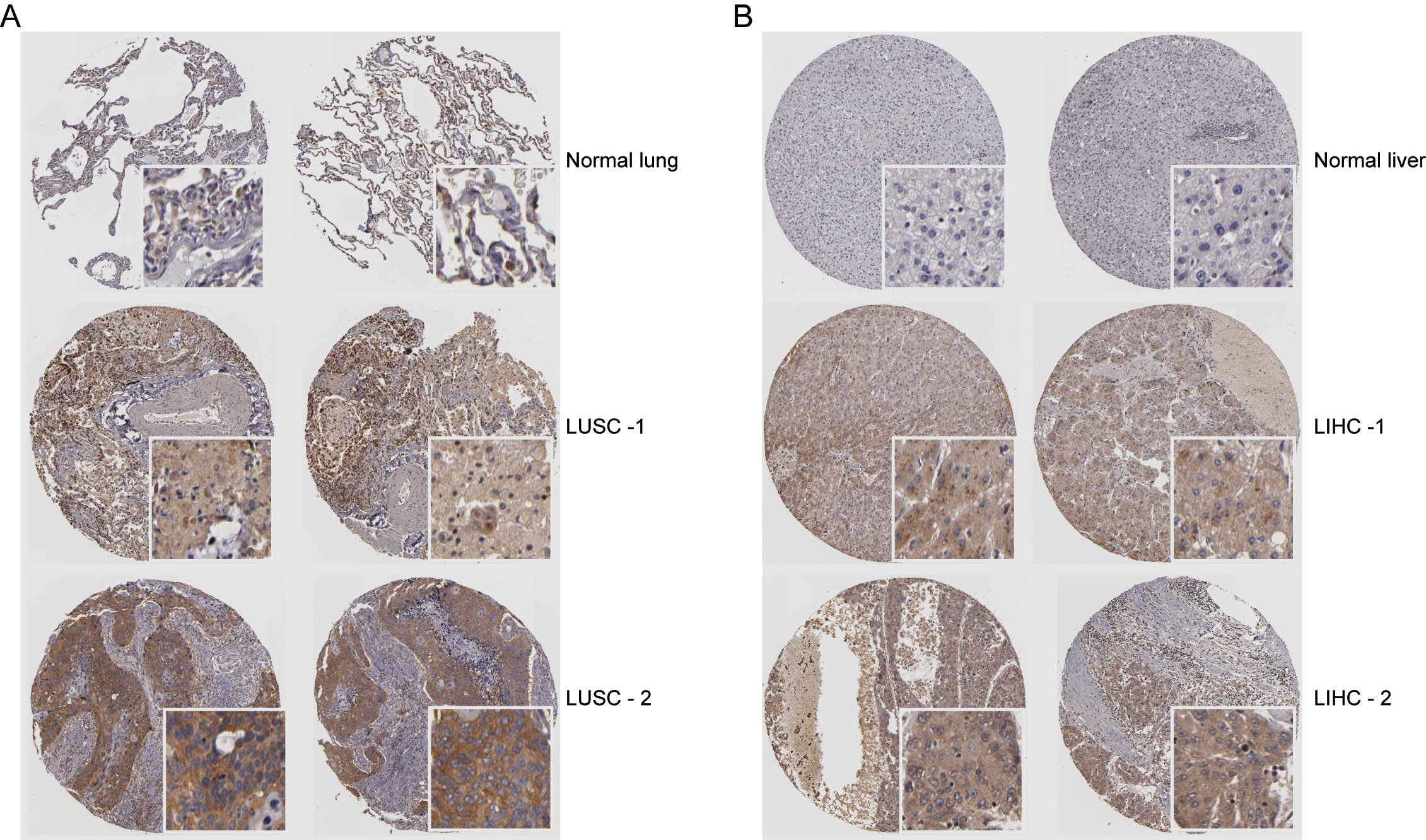

Supplement: Supplementary file 3 — Additional file 3. [file 12672_2024_1369_MOESM3_ESM.tif]

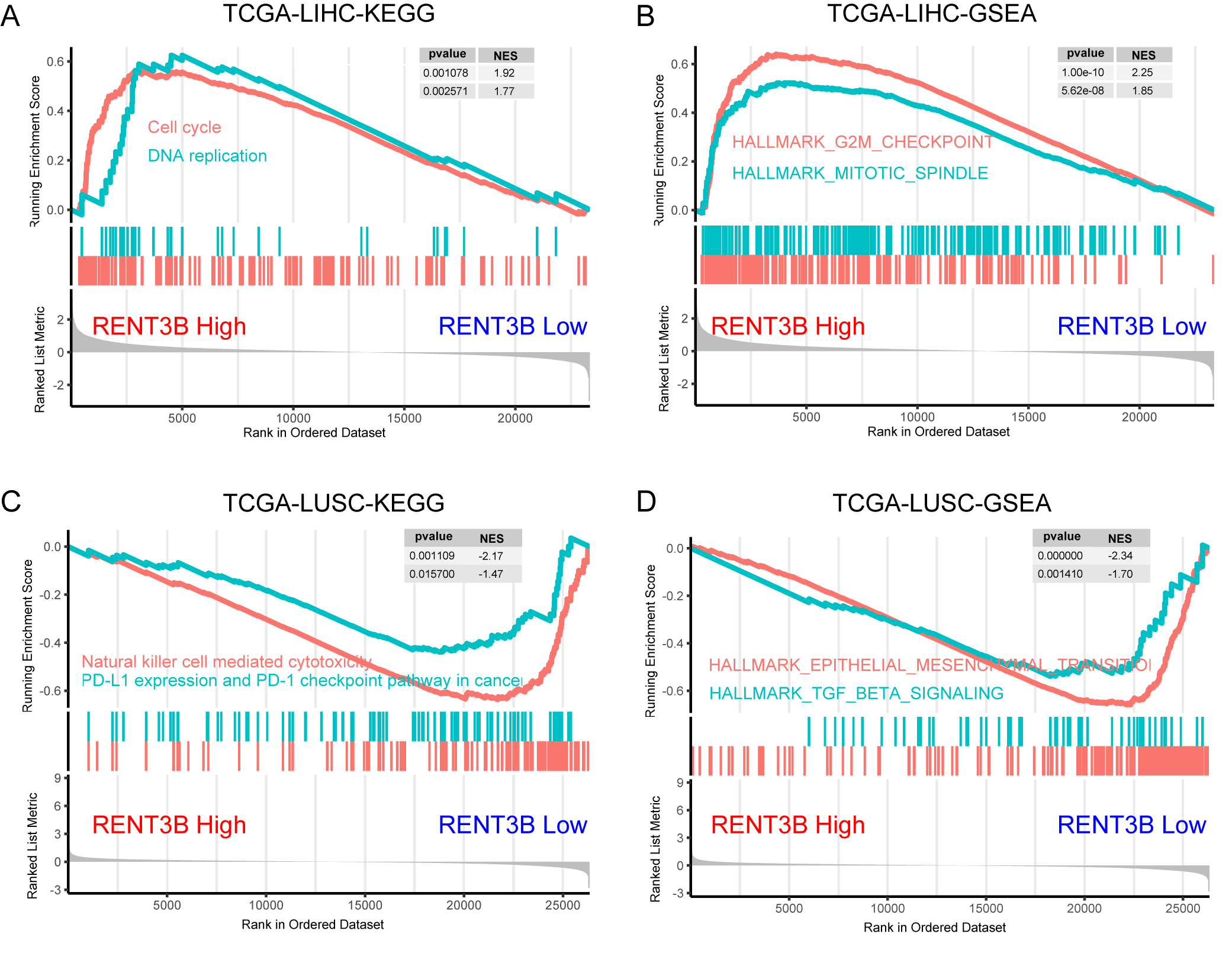

Supplement: Supplementary file 4 — Additional file 4. [file 12672_2024_1369_MOESM4_ESM.tif]
