# Supplementary material for: Regulator of nonsense transcripts 3B is a prognostic biomarker and associated with immune cell infiltration in lung squamous cell and hepatocellular carcinoma
Source: Discov Oncol. 2024 Sep 27;15:479. doi: 10.1007/s12672-024-01369-3 (PMC11436519; doi:10.1007/s12672-024-01369-3)
Supplement: Supplementary file 5 — Additional file 5. [file 12672_2024_1369_MOESM5_ESM.docx]

**Supplementary Table 1. The expression of RENT3B in ONCOMINE database compared with normal tissues.**

| **Cancer** | **Cancer subtype** | ***P*-value** | **Fold**  **change** | **RANK**  **(%)** | **Sample** | **Reference**  **(PMID)** |
| --- | --- | --- | --- | --- | --- | --- |
| Breast Cancer | Medullary Breast Carcinoma | 5.15E-09 | 1.534 | 7% | 1173 | 22522925 |
|  | Ductal Breast Carcinoma | 2.16E-05 | 1.684 | 7% | 1207 | 16473279 |
|  | Invasive Breast Carcinoma | 3.19E-30 | -10.787 | 2% | 211 | 18438415 |
| Cervical Cancer | Cervical Squamous Cell Carcinoma | 2.70E-09 | 2.79 | 1% | 65 | 17974957 |
|  | Cervical Squamous Cell Carcinoma | 1.81E-08 | 1.687 | 2% | 242 | 18506748 |
|  | Cervical Cancer | 3.87E-09 | 3.648 | 3% | 484 | 17510386 |
|  | Cervical Squamous Cell Carcinoma | 3.00E-07 | 1.902 | 5% | 827 | 18191186 |
| Colorectal Cancer | Colon Adenocarcinoma | 9.59E-14 | 1.879 | 1% | 96 | 17615082 |
|  | Cecum Adenocarcinoma | 5.46E-07 | 1.769 | 3% | 530 | 17615082 |
|  | Colon Mucinous Adenocarcinoma | 2.56E-06 | 1.675 | 4% | 640 | 17615082 |
|  | Rectosigmoid Adenocarcinoma | 3.08E-05 | 1.726 | 5% | 847 | 17615082 |
|  | Rectosigmoid Adenocarcinoma | 2.39E-05 | 2.09 | 4% | 692 | TCGA |
|  | Cecum Adenocarcinoma | 6.76E-10 | 2.243 | 5% | 1019 | TCGA |
|  | Rectal Adenocarcinoma | 2.90E-12 | 1.645 | 9% | 1653 | TCGA |
|  | Colon Mucinous Adenocarcinoma | 8.96E-05 | 1.533 | 19% | 3853 | TCGA |
|  | Colon Carcinoma | 2.56E-07 | 2.471 | 7% | 1214 | 20957034 |
|  | Colorectal Carcinoma | 1.39E-06 | 1.528 | 13% | 2516 | 20143136 |
| Esophageal Cancer | Esophageal Squamous Cell Carcinoma | 9.69E-06 | 1.603 | 6% | 727 | 20955586 |
|  | Esophageal Squamous Cell Carcinoma | 1.14E-11 | 1.584 | 6% | 957 | 21385931 |
|  | Esophageal Adenocarcinoma | 1.59E-06 | -1.607 | 19% | 3520 | 21152079 |
|  | Barrett's Esophagus | 7.48E-05 | -1.586 | 22% | 3967 | 21152079 |
| Gastric Cancer | Gastric Intestinal Type Adenocarcinoma | 1.73E-06 | 1.882 | 13% | 2372 | 19081245 |
| Leukemia | B-Cell Acute Lymphoblastic Leukemia | 3.91E-37 | 2.081 | 3% | 409 | 20406941 |
|  | Pro-B Acute Lymphoblastic Leukemia | 2.88E-24 | 1.829 | 4% | 597 | 20406941 |
|  | B-Cell Childhood Acute Lymphoblastic | 1.23E-29 | 1.653 | 6% | 1089 | 20406941 |
| Liver Cancer | Hepatocellular Carcinoma | 9.51E-09 | 2.139 | 2% | 220 | 21159642 |
|  | Hepatocellular Carcinoma | 7.56E-52 | 1.999 | 4% | 440 | 21159642 |
| Lung Cancer | Squamous Cell Lung Carcinoma | 1.17E-06 | 1.767 | 12% | 2241 | 20421987 |
| Lymphoma | Unspecified Peripheral T-Cell Lymphoma | 1.09E-07 | 1.886 | 12% | 2281 | 17304354 |
| Other Cancer | Vulvar Intraepithelial Neoplasia | 1.19E-05 | 2.346 | 2% | 337 | 17471573 |
|  | Embryonal Carcinoma | 7.28E-06 | 2.576 | 11% | 1949 | 16424014 |
|  | Mixed Germ Cell Tumor | 1.09E-05 | 2.222 | 18% | 3176 | 16424014 |
|  | Yolk Sac Tumor | 7.91E-05 | -2.137 | 15% | 2524 | 16424014 |
| Ovarian Cancer | Ovarian Serous Cystadenocarcinoma | 8.86E-07 | 1.956 | 4% | 476 | TCGA |
|  | Ovarian Carcinoma | 3.71E-05 | -1.551 | 16% | 2005 | 18593951 |
| Pancreatic Cancer | Pancreatic Ductal Adenocarcinoma | 4.63E-07 | 1.871 | 14% | 2622 | 19260470 |
| Sarcoma | Pleomorphic Liposarcoma | 9.65E-09 | 2.635 | 3% | 271 | 20601955 |
|  | Myxofibrosarcoma | 3.43E-09 | 2.685 | 5% | 578 | 20601955 |
|  | Leiomyosarcoma | 1.85E-08 | 2.484 | 5% | 595 | 20601955 |
|  | Myxoid/Round Cell Liposarcoma | 6.83E-06 | 1.883 | 14% | 1662 | 20601955 |
